# Investigating the Consequences of the Socio-Instrumental Use of Enterprise Social Media on Employee Work Efficiency: A Work-Stress Environment

**DOI:** 10.3389/fpsyg.2021.738118

**Published:** 2021-08-26

**Authors:** Shuhui Wu, Abdul Hameed Pitafi, Sheena Pitafi, Minglun Ren

**Affiliations:** ^1^School of Management, Hefei University of Technology, Hefei, China; ^2^Department of Business Studies, Bahria University, Karachi, Pakistan

**Keywords:** ESM usage, work efficiency, challenge stressors, hindrance stressors, resilience

## Abstract

Enterprise social media (ESM) has been widely adopted by firms for employee work-related communication. However, it is still debatable how such usage benefits work stress and employee work efficiency. Applying the transactional theory of stress, this study examined the impact of resilience as a moderator on the link between work stressors and individual work efficiency. A total of 285 entries were used to analyze the proposed hypothesis, using structural equation modeling (SEM) technique and hierarchical regression analysis on SPSS 21.0 and AMOS 21.0 software. According to the findings, ESM use has a considerable impact on work stresses (challenge and hindrance). The findings also revealed that challenge stressors have a beneficial influence on employee work efficiency, while hindrance stressors have a negative impact on it. Furthermore, the results also indicated that resilience strengthens the positive relationship between challenge-stressed employees and work efficiency. However, the findings also revealed that resilience reduces the negative association between hindrance-stress and work efficiency. Finally, the author also explored the study's implications for theory and management.

## Introduction

In the contemporary digital work environment, employees frequently use the extensive functions of enterprise social media (ESM), including real-time assistance, video calling, online meeting, and information sharing, to collaborate and interact with workmates (Leonardi and Meyer, 2015). ESM not only permits social interaction, coordination, and the possibility of interpersonal relationships but also assists employees in becoming more productive at work (Treem et al., 2020; Yang et al., 2021). As a consequence, several firms have adopted ESM platforms such as Yammer and Jive for employee communication and collaboration (Song et al., 2019). Research has investigated such popularity of ESM by employees by categorizing its usage as social-related and work-related (Ali-Hassan et al., 2015; Chen and Wei, 2019). The social-related use of ESM reflects its use by individuals for personal and social communication, such as exchanging personal experience, emotions, and suggestions with workmates (Chen and Wei, 2019). The work-related use of ESM refers to employees using it for work-related activities, including clarifying task objectives, planning task assignments, checking work progress, and exchanging work-related information with workmates (Ali-Hassan et al., 2015). Following past research (Ali-Hassan et al., 2015; Wang et al., 2021), the current study refers to the combined work- and social-related use of ESM as socio-instrumental use. Given the touted benefits of ESM usage in the workplace, scholars have investigated its consequences on individual job performance (Pitafi et al., 2018b; Cao and Yu, 2019) and observed mixed results. Accordingly, Pitafi et al. (2018a) observed that ESM usage has a significant effect on employee work performance. In contrast, Cao and Yu (2019) reported that ESM usage is negatively related to employee work performance. The polarization of these statements suggests that more research into the underlying processes through which ESM usage is connected to employee work efficiency is required.

The difference of opinion on the correlation between ESM usage and work efficiency has led to an increased research emphasis on the workplace environment. Furthermore, complicated and unpredictable competing environments are typically described by a variety of work stressors (Islam et al., 2021b; Wang et al., 2021). As a result, managers and academics are beginning to examine the effects of work stressors when formulating techniques for increasing employee efficiency. A “work stressor” is a psychological evaluation of stress connected to specific task stresses and job results (Lazarus and Folkman, 1984; Cavanaugh et al., 2000). Earlier research has revealed that employees may handle some workplace stress, feeling that it is manageable (Cavanaugh et al., 2000; Ding et al., 2019). As a result, dealing with such stresses may have a major impact on individual job efficiency. However, some stressors may be considered unmanageable and these may hinder the potential for individual employee growth (Cavanaugh et al., 2000; Ding et al., 2019; Islm et al., 2021) and have a negative effect on employee work efficiency. Given the distinct outcomes of work stress, managers may experience serious problems in encouraging the work efficiency of stressed employees in particular contexts. Furthermore, prior research has showed that social support is a useful resource that is predicted to boost the motivation and work efficiency of stressed employees (Ali-Hassan et al., 2015; Wang et al., 2021). Employees are also more willing to pursue social support from workmates in the presence of threat or uncertainty in order to determine the appropriateness of their feelings and decrease their levels of anxiety and fear. ESM usage may be effective in enhancing the work efficiency of stressed employees by offering social support (Ali-Hassan et al., 2011, 2015). For example, ESM usage may assist stressed employees to solve work-related issues by strengthening their access to information and their capacity to interconnect with colleagues. As a consequence, the current study investigates the influence of ESM usage on work efficiency through work stressors.

In the literature on stress, resilience is one of the most salient factors affecting how employees react to work stressors (Delany et al., 2015; Kimura et al., 2018). “Resilience” is the ability of employees to adopt, overcome, and develop their potential to effectively deal with challenging situations (Lian and Tam, 2014; Delany et al., 2015). Workplace resilience has been recognized as a crucial, strategically significant organizational behavior for performance, development, and even the ability to survive (Kimura et al., 2018). Furthermore, Lazarus and Folkman (1984) found that workers' self-rated individual characteristics may alleviate the adverse effects of work stress. Similarly, Bakker and Demerouti (2014) observed that, although earlier research has focused on the moderating influence of job resources, the findings regarding the relationship between individual characteristics and work demand is still limited. In addition, personal resources indicate an individual's perception of their capacity to successfully control and influence their surroundings, particularly under difficult circumstances (Hobfoll et al., 2003; Khan et al., 2019). Resilience is described as the capacity to rebound stronger with more capabilities, protecting individuals from emotional damage and enhancing the possibility of resolving stressful situations more effectively. It is thus imperative to hypothesize and investigate the moderating influence of resilience on the link between work stress and employee work efficiency.

This study extends the body of knowledge by investigating (i) how ESM usage influences employees' work efficiency through work stressors, and (ii) the moderating role of resilience on the association between work stressors and employee work efficiency. This research makes several contributions to the theoretical literature. Firstly, existing research examines the link between ESM usage and work efficiency through work stress by categorizing work stress into “challenge” and “hindrance” stress (LePine et al., 2005). ESM usage may benefit stressed employees by providing emotional and social support from managers or workmates (Ali-Hassan et al., 2015). Secondly, this study investigates the role of resilience as a moderator on the link between work stressors and work efficiency. Thirdly, we investigate the distinct impact of both challenge and hindrance stress on employee work efficiency. The results of this study can also guide managers in handling challenge and hindrance stress effectively, as both have a significant impact on employee work efficiency.

## Literature Review and Theoretical Background

### Transactional Theory of Stress

According to the transactional theory of stress, individuals can respond psychologically, behaviorally, and cognitively to stresses that are related to environmental situations—it is a psychological process that connects stressors to work outcomes (Lazarus and Folkman, 1984). The challenge-hindrance model has been proposed to analyze employees' workplace stressors by the transactional theory of stress (Pearsall et al., 2009; Webster et al., 2011). A fundamental approach is that challenge stressors present opportunities for employees which include rewards and personal development (Jex and Bliese, 1999; Cavanaugh et al., 2000). Hindrance stressors inhibit personal development and goals (Wang et al., 2021). Several scholars have recently employed the transactional theory of stress and investigated employees' work outcomes (Webster et al., 2011; Ma et al., 2021; Wang et al., 2021). For example, Wang et al. (2021), using transactional theory, reported that employees' satisfaction with work may mediate the link between hindrance stressors and employee creativity. Webster et al. (2011), using transactional theory to conduct a qualitative investigation, found that challenge-stressed employees have a more significant effect on job performance than hindrance-stressed employees. This study contributes to a transactional theory of stress by leveraging this lens to explain how the social-instrumental use of ESM shapes the influence of work stressors on employee's work efficiency. Using this perspective, we consider the social-instrumental use of ESM as conducive to employees' ability to leverage work stressors to work more efficiently.

In addition, scholars are increasingly considering another crucial assumption of transactional theory: that contextual circumstances can change stress appraisal systems (Cavanaugh et al., 2000; Webster et al., 2011; Ding et al., 2019). By applying transactional theory, researchers have found certain characteristics that might increase the probability that workers can deal efficiently with challenge stresses and decrease the tendency of individuals to evaluate the possible effects of hindrance stressors (Häusser et al., 2010; Wang et al., 2021). For example, Jex and Bliese (1999) observed that the link between work stressors and outcomes may be moderated by several factors that include the workplace environment, demographic characteristics, and personal traits. As consequence, we use transactional theory to propose that the resilience behavior of employees may moderate the association between work stressors and employee work efficiency. Previous studies have also reported that social support from colleagues may influence individuals' psychological responses to stressors (Ding et al., 2019). As a result, ESM usage has the potential to benefit individuals' processes of stress appraisal.

### Work Stressors

Work stressors develop from workplace responsibilities that might cause stress and associated behavioral mechanisms (Lin W. et al., 2015). Its principal categories are challenge stressors and hindrance stressors (Cavanaugh et al., 2000). Challenge stressors are workplace requirements that are challenging but achievable; employees can overcome them to achieve their job objectives and develop competency (Wallace et al., 2009). Examples of challenge stressors include occupational stress, time pressure, task difficulty, and a high level of responsibility. Hindrance stresses are occupational factors that employees perceive as needless barriers to overall achievement and personal development and achievement (Wallace et al., 2009; Ding et al., 2019). Examples included role conflict, role uncertainty, institutional limitations, personal problems, and unsure job security. Challenge stressors can enhance individual learning and goal attainment, motivating them to use creative problem-solving and coping mechanisms (Fay and Sonnentag, 2002). On the other hand, hindrance stressors have a negative effect on job outcomes (Wallace et al., 2009).

Existing research on the influence of challenge and hindrance stressors on individual work performance has inconsistent results (Jex and Bliese, 1999; Wallace et al., 2009). For example, Wallace et al. (2009) observed that challenge stressors have a significant impact on work outcomes, while hindrance stressors have an adverse effect on them. Similarly, Wang et al. (2021) found that challenge stressors are significantly linked to employee creativity while hindrance stressors are negatively related. LePine et al. (2005) conducted a meta-analysis and observed that challenge stressors have a significant impact on job performance whereas hindrance stressors have a negative impact. Theoretical literature reflects that, overall, challenge stressors are beneficial whereas hindrance stressors are not. In order to reap the benefits of challenge stressors and mitigate the adverse effects of hindrance stressors, scholars have examined different moderators such as management support (Wallace et al., 2009), social support, and social media usage (Ding et al., 2019; Wang et al., 2021). Nevertheless, no empirical research has been conducted to evaluate the possible moderating impact of employee resilience on the link between work stressors and work efficiency.

### Socio-Instrumental Use of ESM

The socio-instrumental use of ESM technology refers to the use of ESM for socialization, the exchange of work-related information, and social support (Zhong et al., 2012; Leonardi and Meyer, 2015; Nand et al., 2019). ESM is a digital network that allows employees to develop social relationships, share knowledge, and provide online assistance (Van Zoonen et al., 2017; Wei et al., 2020). For example, ESM can highlight individuals' interests and hobbies, thus encouraging the formation of social networks among coworkers. Specifically, ESM employs a social tagging mechanism to track who bookmarks information on specific expertise; it thus assists workers to identify who is proficient in specialized knowledge and skill inside their enterprise. ESM usage not only offers the possibility of social relationships and interaction but it also improves employees' work performance (Cao et al., 2016; Latif et al., 2021). Existing work on ESM has shown that its use promotes employee agility performance (Islam et al., 2020; Pitafi et al., 2020b), creativity (Chen et al., 2020; Nadeem et al., 2021), innovation, and knowledge-sharing (Engelbrecht et al., 2019). ESM has been adopted by an increasing number of businesses to improve employee performance since, as a socializing platform, it encourages employee engagement, the establishment of mutual understanding, and the formation of online communities (Chen and Wei, 2019; Wu et al., 2020). For example, ESM tools such as Yammer, DingTalk, and Jive have been widely implemented by several companies to support employees' work-related communication and collaboration. The popularity of ESM not only changes workplace communication and collaboration but also presents significant possibilities for stress management and work performance (Cai et al., 2018; Cao et al., 2020).

In addition, ESM usage also effects employee cognition, psychological states, and job engagement. For instance, Cai et al. (2018) observed that ESM use in the workplace may benefit employee agility through psychological conditions. Lin T.-C. et al. (2015) proposed that employees may obtain social support from workmates on ESM platforms. While Van Zoonen et al. (2017) argued that using ESM might cause individual exhaustion from too much disruption, research in this domain is inadequate. Moreover, little research has explored the possible role of social media use in stressor consequences (Ding et al., 2019; Islam et al., 2021c; Wang et al., 2021). Nonetheless, it is still not known how the use of social media may influence employees' evaluation of stress and attitudinal responses to it. Consequently, existing research has examined the impact of ESM on employee efficiency through work stressors.

## Hypothesis Development

### Socio-Instrumental Use of ESM, Work Stressors

According to prior literature, individuals' stress appraisal processes are affected by ESM usage (Ding et al., 2019; Wang et al., 2021). The socio-instrumental use of ESM offers social support—including work-related assistance, information, and suggestions—which can enable employees to complete tasks on time (Ali-Hassan et al., 2015; Ding et al., 2019; Rasheed et al., 2020b). Challenge-stressed employees are thus motivated to perceive their tasks as manageable (Wallace et al., 2009; Rasheed et al., 2020a). By using ESM, employees can share all task-related issues, expertise, plans, and statuses with workmates (Pitafi et al., 2019; Rashid et al., 2020). Such task-related information challenges stressed employees to obtain important information and manage their tasks efficiently. ESM usage could benefit challenge-stressed individuals to gain insight into the possible advantages of tasks, such as promotion or self-development (Cai et al., 2018; Anser et al., 2020; Wang et al., 2021). In addition, ESM usage promotes a strong perception of stressful demands to challenge-stressed individuals, thereby enhancing their willingness to spend their time and energy resolving task-related issues (Ding et al., 2019; Nadeem et al., 2020). The socio-instrumental use of ESM also provides social support which can assist stressed employees to develop positive responses to stressful demands (Ali-Hassan et al., 2015; Chen et al., 2018). Therefore, with a socio-instrumental use of ESM, challenge-stressed workers may believe that they might have task-related sources to plan their tasks and that they can efficiently solve task-related problems with social support from their managers. In light of the above discussion, we propose the following hypothesis:

H1a: Socio-instrumental use of ESM has a negative effect on challenge stressors.

The effect of the socio-instrumental use of ESM on hindrance-stressed employees is same as for challenge-stressed employees. The higher the use of ESM, the more resources and social support that are available for hindrance-stressed employees. In particular, hindrance-stressed employees focus on cognition and psychological separation from other workmates because they develop negative feelings and motivations (Cavanaugh et al., 2000; Wallace et al., 2009; Islam et al., 2021a). ESM usage among employees may stimulate hindrance-stressed employees to be less worried about task-related issues and job security when dealing with task-related problem-solving (Webster et al., 2011; Treem et al., 2020; Lai et al., 2021). In this situation, hindrance-stressed individuals may encounter fewer situational hurdles in attempting multiple methods and can clearly explain uncertainties by frequent communication and problem-solving. ESM usage thus promotes job control mechanisms, which may reduce the adverse impact of hindrance stressors and encourage workers to discuss and use different approaches to solving problems (Khan et al., 2020; Yang et al., 2021). In addition, social support may assist hindrance-stressed employees to develop significant emotional responses to deal with stressful demands (Treem and Leonardi, 2013; Ali-Hassan et al., 2015). The socio-instrumental use of ESM facilitates employees in acquiring social and task-related support from their workmates or managers within the organization (Chen and Wei, 2019; Pitafi and Ren, 2021) which, in turn, reduces their fear and anxiety. As a result, when hindrance-stressed workers use ESM, they may be guided by other workers while analyzing the hindrance scenario (Engelbrecht et al., 2019; Latif et al., 2020); ESM usage thereby lowers the perception of threat. In summary, ESM usage promotes the social support which may weaken the negative perception of hindrance stressors. Hence, this study proposes the following hypothesis:

H1b: Socio-instrumental use of ESM has a negative effect on hindrance stressors.

### Work Stressors, Employee Work Efficiency

Work stressors are causes of stress in the workplace that are often considered detrimental in an organization because they are related to individuals' psychological, physical, and cognitive strain (Pearsall et al., 2009; Webster et al., 2011). In recent decades, scholars have claimed that some workplace stresses can be beneficial to work outcomes (Webster et al., 2011; Pitafi et al., 2020b; Wang et al., 2021). Research has shown challenge stressors to be significantly associated with work outcomes, whilst hindrance stressors were shown to be negatively associated with them (LePine et al., 2005; Wallace et al., 2009). Nevertheless, research on the association between workplace stressors and work performance has yielded conflicting or inconclusive results (Jex and Bliese, 1999). An emerging body of research recognizes that the adoption of IT has a wide and indirect impact on employee work outcomes (Pitafi et al., 2018b). As a result, the objective of existing research is to explore the effects of workplace stressors on employee work efficiency in an ESM context.

Challenge-stressed employees can boost individuals' knowledge and goal attainment by motivating them to engage in active problem-solving and coping mechanisms (González-Morales and Neves, 2015; Kim and Beehr, 2020). According to research, challenge stresses are significantly correlated with individuals' work motivation (Fay and Sonnentag, 2002; Liu et al., 2013). When workplace stressors are considered controllable, they may elicit positive behaviors from workers, such as attention, excitement, and confidence (Wallace et al., 2009; Wang et al., 2021); this engages them in critical problem-solving. Hence, when dealing with challenging tasks, challenge-stressed individuals will use several strategies to solve problems, having a significant effect on work efficiency. Accordingly, time or work pressure may encourage employees to completely concentrate on their tasks (González-Morales and Neves, 2015; Kim and Beehr, 2020), thereby improving employee job efficiency. On the other hand, hindrance stressors lead to a passive style of survival mechanism, such as reducing one's work efforts (Ding et al., 2019). Hindrance stressors are unmanageable and thus have negative effects on employees, such as anxiety, fear, and frustration (Cavanaugh et al., 2000; Wallace et al., 2009). Prior study has also reported that hindrance stressors have negative impacts on work motivation (Kim and Beehr, 2018; Ali et al., 2019). Accordingly, hindrance-stressed workers are more concerned about their job security and are less focused on their work (Webster et al., 2011), thereby negatively impacting work efficiency. In such a case, a stressed individual may not make any additional effort to solve work-related challenges. Hindrance stressors may also inhibit the possibilities of individual development, causing workers to withdraw from an existing situation (Wallace et al., 2009). Such negative motivation may discourage employees from problem-solving and have a negative effect on work efficiency. On the basis of literature review, this study proposes the following hypotheses:

H2a: Challenge stressors have a positive effect on employee work efficiency.
H2b: Hindrance stressors have a negative effect on employee work efficiency.

### Role of Resilience as a Moderator

As mentioned above, challenge and hindrance stressors have different impacts on work efficiency. Accordingly, we further assume that resilience may moderate the link between challenge stressors, hindrance stressors, and work efficiency. The concept of resilience reflects the ability or dynamic process of positive adjustment in the face of danger that may be achieved through specific environmental conditions (Crane and Searle, 2016; Foster et al., 2020). The transactional theory of stress proposes that efficiently dealing with stressful situations may assist individuals in enhancing their resources and work efficiency (Kanwal et al., 2019d; Wang et al., 2021). Indeed, with resilience, challenge stresses may be overcome by individual effort and have positive results that may strengthen individuals' trust and capacity to adapt to future problems (Friborg et al., 2006; Hao et al., 2015). Scholars reported that resilience is the ability to recover or bounce back from stress and use it as a tool for managing a challenging job (Avey et al., 2009; Crane and Searle, 2016). Studies have also found that higher resilience has a significant effect on job satisfaction, task engagement, and work performance (Hartmann et al., 2020; Jannesari and Sullivan, 2021). Hao et al. (2015) observed that highly resilience personalities are more confident and capable of responding to challenge stress, and they are more efficient at utilizing surrounding supportive resources. Hence, resilience benefits challenge-stressed employees in coping with challenges more efficiently.

Furthermore, we propose that resilience alleviates the negative association between hindrance-stressed individuals and work efficiency. Scholars suggest that resilience promotes work performance, making a greater impact on well-being and reducing the negative outcome of stressors (Cole et al., 2015; García-Izquierdo et al., 2018). Resilience promotes individuals' capacity to recognize, analyze, and respond to overwhelming events, such as hindrance stress in the workplace (Heath et al., 2020). Resilience thus assists individuals to avoid negative encounters and respond to stress effectively. Hence, it can enhance an overall significant effect on employees and reduce strain frequency (Crane and Searle, 2016; Kimura et al., 2018; Annor and Amponsah-Tawiah, 2020). Individuals who consider themselves resilience are more likely to believe that they can cope with various pressures (Naz, 2020; Bani-Melhem et al., 2021). Consistent with this statement, prior research reflects resilience as having a negative impact on work-related anxiety and emotional exhaustion (Bande et al., 2015; Kossek and Perrigino, 2016). As a result, individuals are more likely to be able to focus their attention on their tasks without feeling anxiety. Thus, the following hypotheses are proposed in this study:

H4a: Resilient behavior by employees moderates the relationship between challenge stressors and employee work efficiency such that the relationship is stronger at a higher level of resilience.
H4b: Resilience behavior by employees moderates the relationship between hindrance stressors and employee work efficiency such that the relationship is weaker at a higher level of resilience.

## Research Methods

### Data Collection Procedures

In order to accomplish the goal of this research, we employed a survey methodology to collect data from Chinese employees. Previous researchers have preferred the survey technique over case studies or experiments because it captures the essence of working environments without compromising generality (Cai et al., 2018; Kanwal et al., 2020a,c). This study was performed in eastern China, the country's most developed area. The author firstly visited several organizations to determine their working environments and ESM usage. The nine organizations included in this study are from the banking and financial sector, service industries, garment industry, and the electronics manufacturing industry. The author then conducted several meetings with managers and discussed the objective of the study. Managers and employees were informed that their feedback would be used for research purposes and that their responses would be kept confidential. Before data collection, the author designed the survey items and invited five faculty members and three Ph.D. students from the information and management department for critical review and suggestions. After discussion, the survey items were slightly modified by incorporating their suggestions. The entire survey consisted of three sections. Section 1 included a cover letter, the objective of the study, and basic concepts about the measurement items. Section 2 captured demographic information about the respondents. The last section comprised the actual measurement items. Before collecting large-scale data from employees, the author conducted a pilot study on 51 respondents and found satisfactory results, motivating further data collection.

The survey was in hard-copy and was collected from ESM users from August to November 2020. A total of 400 questionnaires was circulated and 315 completed questionnaires were received within 3 months, with a response rate of (78.75%). However, 30 questionnaires were eliminated as being improperly completed or with some entries left blank. The final data set this consisted of 285 responses. The information of respondents surveyed is shown in Table 1.

**Table 1:** Demographics.

**Variables**	***N***	**Percentage**	**Variables**	***N***	**Percentage**
**Gender**			**Qualification**		
Male	180	63.20	Under-graduate	42	14.70
Female	105	36.80	Graduate	125	43.90
**Age**			Masters or Above	118	41.40
Between 21–30	95	33.30	**Experience**		
Between 31–40	126	44.20	<1 year	20	7.00
More than 41	64	22.50	2–3 years	29	10.20
			4–5 years	40	14.00
			More than 5 years	119	41.80

### Research Instrument

We developed all the instruments and measurements from previous related studies, which were published in well-reputed journals. All employees in this study were Chinese, so we followed the procedure of previous studies (van de Vijver and Leung, 1997; Pitafi et al., 2018a) and invited three native Chinese interpreters to translate the original English language questionnaire into Chinese. The author then invited another five native Chinese experts who were fluent in English to translate the Chinese language questionnaire into English. On comparing the original questionnaire and the translated version, we found no semantic difference between the translated English version and the original; this indicated that the Chinese items properly matched the original English meaning. Therefore, a Chinese questionnaire was used for data collection. All the measurement items were computed using a five-point scale from “strongly agree” to “strongly disagree.” Details of all the instruments are below.

#### Challenge and Hindrance Stressors

The measurement items of challenge and hindrance stressors were measured using Cavanaugh et al. (2000) and Ding et al. (2019). Five items were used to compute the challenge stressors; an example is “In my organization, I experience time pressure.” Hindrance stressors were computed using five items; for example, respondents were asked to respond to, “I have a lack of job security in my organization.”

#### Work Efficiency

The outcome variable of work efficiency included eight items, and was borrowed from Janssen and Van Yperen (2004) and Yang et al. (2021). An example from the questionnaire about work efficiency is, “I perform better than my colleagues.”

#### Socio-Instrumental Use of ESM

To compute the socio-instrumental use of ESM, we borrowed eight items of Zhong et al. (2012). An example item of this scale is “I am often involved with coworkers for receiving or sending technical assistance via an ESM network.”

#### Resilience

We used resilience as a moderator in this research study; it consisted of eight items which were borrowed from Smith et al. (2008). An example item of the resilience scale is “I can perform my job efficiently in difficult or stressful situations.”

#### Control Variables

To measure the actual effect of independent variables on a dependent variable, this study considered respondents' gender, age, education level, and experience as control variables (Kanwal et al., 2020b).

## Results and Analysis

We used SPSS and AMOS software to perform the regression and structural equation modeling to analyze the proposed hypotheses and research model. For data analysis, we initially screened 285 entries on SPSS software, observing nothing missing or any outliers in the data set. All the analysis was conducted in three phases. Firstly, the author analyzed the validity, reliability, and factor loading of all the constructs. In the second phase, the author applied structural equation modeling to analyze the relationship among constructs. Finally, regression analysis was used for moderation analysis.

### Instrument Validation

Following guidance from previous studies, we also computed the convergence, content, and reliability of measurement items using several statistical techniques (Fornell and Larcker, 1981; Hinkin, 1998; Kanwal et al., 2019e). Accordingly, the convergent validity of the research model was evaluated using three indicators: Cronbach's α (CA), composite reliability (CR), and average variance extracted (AVE), as suggested by Fornell and Larcker (1981). The results in Table 2 indicate that CA values range (0.818–0.880) and that the range of CR values (0.857–0.891) is higher than the minimum suggested value of 0.700. Similarly, AVE values, ranging (0.516–0.711), are higher than the minimum suggested value of 0.500, as shown in Table 2 (Bagozzi et al., 1991; Kanwal et al., 2019b). The findings of **Table 4** also indicate that the standard loading of all the measurement items is higher than the suggested value of 0.600 (Fornell and Larcker, 1981). These results indicated that the suggested research model has an acceptable level of convergent validity and reliability.

**Table 2:** Results of measurement analysis.

**Constructs**	**Items**	**Cronbach α**	**Composite reliability**	**AVE**
Resilience	6	0.880	0.882	0.528
Socio-instrumental use of ESM	6	0.818	0.870	0.711
Perceived challenge stressors	6	0.821	0.864	0.516
Perceived hindrance stressors	6	0.823	0.857	0.600
Work efficiency	7	0.871	0.891	0.543

*AV, Average Variance Extracted (AVE)*.

After convergent validity and reliability, the author also investigated the discriminant validity of the research model using distinct procedures. Firstly, we followed Fornell and Larcker (1981) in assessing the discriminant validity of the research model. According to this procedure, we compared the square root of AVE with pairwise inter co-relation values of each construct with AVE square roots in Table 3. The results of this observation indicate that all AVE square root values are higher than the inter co-relation values of all the constructs, indicating an acceptable level of discriminant validity of the research model. Secondly, we also observed the findings of Table 4, which reflected that the values of all the constructs of all items were loaded higher in their respective columns and poorly loaded into other columns. As a consequence, we surmised that the suggested conceptual model has an adequate level of discriminant validity.

**Table 3:** Correlation matrix and Mean, Standard Division.

**Construct**	**Mean**	**SD**	**1**	**2**	**3**	**4**	**5**	**6**	**7**	**8**	**9**
1. Resilience	3.524	0.982	**0.726**								
2. Socio-instrumental use of ESM	2.014	0.638	−0.062	**0.843**							
3.Perceived challenge stressors	3.537	0.720	0.444^**^	−0.255^**^	**0.718**						
4.Perceived hindrance stressors	2.155	0.730	−0.616^**^	0.056	0.352^**^	**0.774**					
5– Work efficiency	3.9530	0.600	0.326^**^	−0.479^**^	0.377^**^	−0.249^**^	**0.736**				
6– Experience	**NA**	**NA**	−0.013	−0.010	−0.013	0.012	−0.020	**NA**			
7- Education	**NA**	**NA**	0.015	−0.048	0.009	−0.071	−0.029	−0.052	**NA**		
8– Age	**NA**	**NA**	−0.092	0.098	−0.014	0.069	−0.040	0.065	−0.039	**NA**	
9- Gender	**NA**	**NA**	0.017	0.100	0.016	−0.012	−0.026	−0.021	−0.011	-0.042	**NA**

*p < 0.05,

** *p < 0.01. Diagonal values indicates the square root of AVE*.

**Table 4:** Cross-loading.

**Construct**	**Items**	**WEF**	**RES**	**HST**	**CST**	**SESM**
Work efficiency (WEF)	WEF01	**0.894**	0.043	0.153	0.016	0.080
	WEF02	**0.799**	−0.018	−0.050	−0.018	−0.021
	WEF03	**0.759**	−0.073	0.054	0.041	−0.004
	WEF04	**0.746**	0.114	0.082	−0.068	−0.058
	WEF05	**0.731**	0.079	−0.121	−0.061	−0.017
	WEF06	**0.671**	−0.045	−0.247	0.050	0.026
	WEF07	**0.617**	−0.028	0.090	0.151	−0.135
Resilience (RES)	RES01	−0.018	**0.870**	0.033	−0.065	0.002
	RES02	−0.026	**0.801**	0.004	0.015	0.010
	RES03	0.019	**0.788**	−0.089	−0.062	−0.044
	RES04	0.108	**0.733**	−0.012	−0.004	0.029
	RES05	0.020	**0.714**	0.024	0.109	0.040
	RES06	−0.018	**0.646**	−0.141	0.099	−0.060
Hindrance stressor (HST)	HST01	−0.133	−0.043	**0.766**	0.087	−0.143
	HST02	0.151	0.071	**0.745**	−0.060	0.123
	HST03	0.114	−0.137	**0.739**	0.028	0.017
	HST04	−0.064	0.054	**0.714**	0.124	0.109
	HST05	0.076	−0.043	**0.621**	−0.236	−0.072
	HST06	−0.087	−0.102	**0.621**	0.069	0.001
Challenge stressors (CST)	CTS01	0.030	−0.076	−0.155	**0.743**	0.049
	CTS02	−0.111	0.053	−0.089	**0.740**	−0.071
	CTS03	0.084	−0.094	0.132	**0.736**	0.003
	CTS04	−0.030	0.079	0.151	**0.729**	0.034
	CTS05	0.142	−0.008	−0.099	**0.675**	0.191
	CTS06	−0.033	0.113	0.094	**0.673**	−0.218
Socio–instrumental use of ESM	SESM01	−0.069	−0.020	−0.138	0.083	**0.766**
	SESM02	−0.011	0.043	0.100	0.029	**0.765**
	SESM03	0.154	−0.101	0.088	−0.011	**0.727**
	SESM04	−0.016	−0.089	−0.216	−0.013	**0.715**
	SESM05	−0.132	0.090	0.216	0.077	**0.654**
	SESM06	−0.092	0.129	0.091	−0.179	**0.624**

*Bold values indicates the highest factor loading of constructs*.

### Common Method Variance (CMV)

Since we collected all the data from a single source using the same procedure, there might be a possibility of CMV in the current study (Podsakoff et al., 2012; Kanwal et al., 2019c). To address the possibility of CMV, we applied several approaches as suggested by previous studies (Podsakoff et al., 2003; Liang et al., 2007). Firstly, we used some procedural methods to overcome the possibility of CMV at the time of data collection. Accordingly, we reversed one item to secure participants' attention while they responded to the survey. Secondly, we applied the common latent factor (CLF) approach to address the possibility of CMV (Podsakoff et al., 2012; Latif et al., 2019). We computed the regression values of all the constructs with and without a common latent factor. By comparing the regression values of both the analyses, we did not find any dominant factor appearing from both. Secondly, we used the procedure suggested by Liang et al. (2007); the results of this analysis indicated that substantive factor loading has 66% of the variance, whereas method factor loading has 1.4% of the variance, thereby indicating that CMV does not occur in this study. Thirdly, we also evaluated the variance inflation factor (VIF); this analysis indicated that VIF scores were lower than the proposed value of 3.3 (Pitafi et al., 2020d), confirming that CMV is not a major issue in the existing data set. As a consequence, all of the information presented above revealed that CMV had no effect on the outcome of this study.

We also assessed this study's research model using the AMOS tool with a maximum likelihood method. Following the guidelines of Hair et al. (2016), the author initially examined the measurement model fit values; the results also indicate that measurement model fit values are in an acceptable range (CFI = 0.893, TLI = 0.879, IFI = 0.894, NFI = 0.812, AGFI = 0.849, REMSA = 0.062, CMIN/DF = 839.17/411 = 2.042), as shown in Table 5.

**Table 5:** Comparison measure model and structural model.

	**Absolute fit measures**			**Incremental fit measures**		**Parsimonious fit measures**		
**Model**	**X ^**2**^/DF**	**SRMR**	**RMSEA**	**NFI**	**PNFI**	**CFI**	**IFI**	**TLI**
MM	2.042	0.061	0.062	0.812	0.849	0.893	0.894	0.879
SEM	2.665	0.074	0.077	0.801	0.846	0.852	0.854	0.830

### Structural Modeling

**Figure 1:**
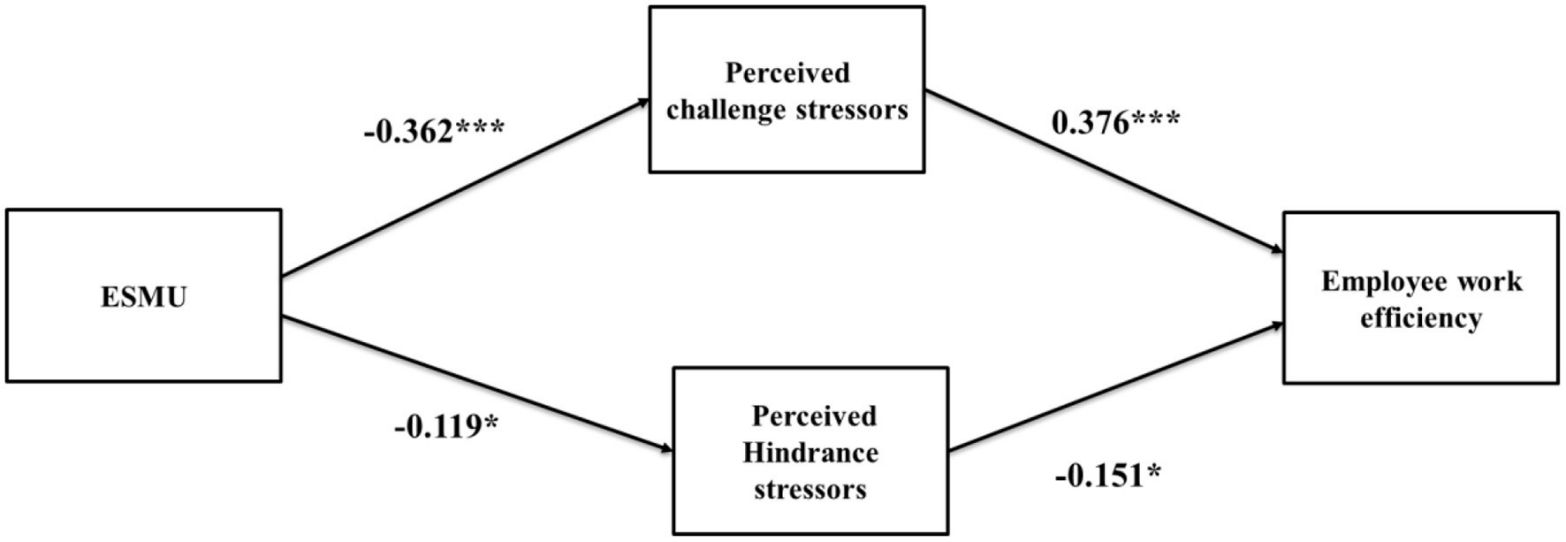
Structural model. ^*^p < 0.05, ^**^p < 0.01, ^***^p < 0.001.

The findings of Table 5 indicate that the model fit values of the structural model (CFI = 0.852, TLI = 0.830, IFI = 0.854, NFI = 0.801, AGFI = 0.846, REMSA = 0.077 CMIN/DF = 695.48/261 = 2.665) are in the specified range (Hair et al., 2017; Kanwal et al., 2019a). Furthermore, Figure 1 shows the results of path analysis of the suggested model. The findings indicate that socio-instrumental use of ESM has a negative effect on challenge stressors (B = −0.362, *t* = −4.38, *p* < 0.001) and hindrance stressors (B = **–**0.119, *t* = 2.01, *p* < 0.05); therefore, both h1a and h1b are accepted. Moreover, the results also showed that challenged stressors have a positive effect on work efficiency (B = 0.376, *t* = 4.93, *p* < 0.001), whereas hindrance stressors have a negative effect (B = **–**0.151, *t* = **–**2.11, *p* < 0.05) on it.

### Moderation Analysis

**Figure 2:**
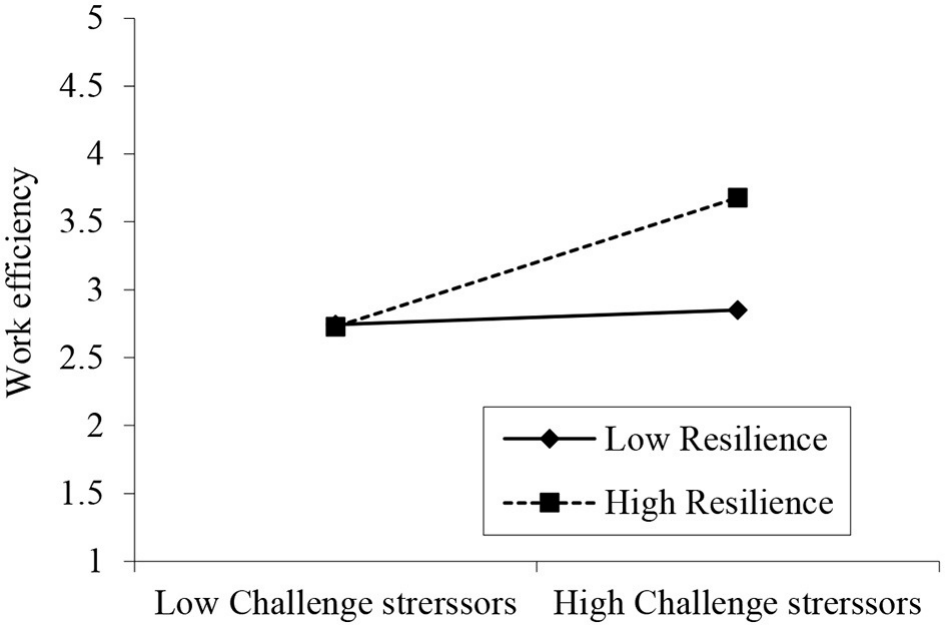
Moderating effect of Resilience in the relationship between Challenge Stressors and Work Efficiency.

In addition, we used hierarchical regression analysis to test the moderating effect of resilience on the relationship between work stressors and work efficiency. To minimize the possibility of multicollinearity, we firstly standardized all the items of all constructs (Aiken and West, 1994). As shown in Table 6, we estimated four hierarchical regressions for the dependent variable work efficiency. In Step 1 (Model 1), the control variables were regressed with work efficiency, revealing that the control variables are not significant. In Step 2 (Model 2), work stressors (challenge/ hindrance) have been entered; the outcome reveals that challenge stressors have a significantly positive effect (β = 0.344, *p* < 0.001) and that hindrance stressor have a negative effect (β = **–**0.146, *p* < 0.01) on work efficiency, supporting h2a, and h2b. In Step 3, resilience was entered in Model 3; the results showed a positive effect for resilience (β = 0.189, *p* < 0.001). Finally, in Step 4, the interaction terms (challenge stressor^*^resilience) and (hindrance stressor^*^resilience) were entered in Model 4. The findings of Model 4 confirmed that resilience reinforced the link between challenge stressors and work efficiency (B = 0.211, *p* < 0.01); therefore, h3a is accepted. In addition, the results also indicated that resilience did not moderate the negative link between hindrance stressors and work efficiency (B = 0.032, *p* > 0.05); therefore, h3b is rejected by the current study. In summary, resilience positively moderates the relationship between challenge stressors and employee work efficiency. As demonstrated in Figure 2, the moderating influence of resilience on the connection between challenge stressors and work efficiency was displayed with standard deviation (+1SD/−1SD) to indicate the influence of a high vs. low degree of each.

**Table 6:** Moderation analysis.

**Variable**	**Model 1**	**Model 2**	**Model 3**	**Model 4**
**Regression analysis**				
Gender	−0.036	−0.107	−0.119^*^	−0.103
Age	−0.053	−0.023	−0.024	−0.060
Education	−0.065	−0.048	−0.038	−0.061
Experience	−0.021	0.030	−0.043	−0.055
**Main effects**				
Hindrance stressors		−0.146^**^	−0.110^*^	0.173^*^
Challenge stressors		0.344^***^	0.298^***^	0.265^***^
**Moderator**				
Resilience			0.189^***^	−0.203^**^
**Interactions**				
Challenge Stressors * Resilience				0.211^**^
Hindrance Stressors * Resilience				0.032
R^2^	0.005	0.170	0.190	0.221
Adjusted R^2^	0.009	0.153	0.170	0.195
F Change	0.357	27.669	6.757	5.361

* *p < 0.05*,

** *p < 0.01*,

*** *p < 0.001*.

## Discussion, Implications, Limitations

### Discussion

The proposed study examined the effects of the socio-instrumental use of ESM on employee work efficiency through work stressors such as challenge and hindrance stressors. On the basis of the transactional theory of stress, this research also examined the moderating role of resilience on the relationship between work stressors and employee work efficiency, using data collected from China. The findings revealed that ESM usage (h1a, h1b) had a negative effect on both challenge and hindrance stressors, implying that both hypotheses are accepted by the current investigation. As with previous research (Ding et al., 2019; Pitafi et al., 2020a; Wang et al., 2021), these results indicate that ESM usage provides the social support, communication visibility, interaction, and work-related information for workers which may reduce the negative effect of work stressors (challenge, hindrance). Evidence also suggests that employees who have greater social support may be capable of dealing with stressful circumstances more effectively (Parrish Meadows et al., 2011). For example, Wang et al. (2021) found that ESM usage moderates the relationship between role conflict, ambiguity, and employee creativity. Similarly, Pitafi et al. (2020c) also reported that ESM usage may benefit workplace conflict, minimize the negative outcome of workplace conflict, and provide social support. The outcome of this study also indicates that challenge stressors have a significant effect on work efficiency; h2a is thus supported. The findings of this study also indicate that hindrance stressors have a negative effect on work efficiency; h2b is thus validated by current data set. Previous scholars also found similar results (Liu et al., 2013; Ding et al., 2019). Accordingly, Ding et al. (2019) found that challenge stressors have a significant impact on creativity while hindrance stressors have a negative effect on it. Similarly, Liu et al. (2013) discovered that challenge stressors improve job performance whereas hindrance stressors had the opposite effect.

Furthermore, this study found that the association between challenging stressors and employee work efficiency is attenuated by resilience—h4a. Previous research has also found that employees with better resilience may respond to stressful circumstances more proactively (Cavanaugh et al., 2000; Parrish Meadows et al., 2011). According to research, resilience may play an important role in managing workplace challenges and coping with stressful occurrences (Sołtys and Wozniewicz, 2015; Younis et al., 2020), and therefore resilient employees are less likely to feel stressed in the presence of work pressure; as a result, they perform better. However, the results showed that resilience did not moderate the negative link between hindrance stresses and work efficiency; hence, h4b is rejected. These findings indicate that, although resilience may be used to effectively cope with stress, it is insufficient for dealing with hindrance stress. This finding suggests that resilience cannot benefit the negative link between hindrance stressors and work efficiency. However, hindrance stressors considerably reduce employees' work efficiency. The demotivation associated with hindrance stress cannot be reduced by resilience and, therefore, it prevents an individual from paying sufficient attention to work.

### Theoretical Implications

The present study also makes a theoretical contribution to the extant literature. Firstly, it investigated the association between ESM usage and employee work efficiency through work stressors, although researchers have observed that individual work efficiency is still in its early stages and is mostly undertaken in workplace settings (Janssen and Van Yperen, 2004; Yang et al., 2021). Despite the prominence of ESM usage, the present research on how employees successfully work when faced with workplace stress in an ESM environment is insufficient and scattered. To address this research gap, this research established and evaluated an IT-dependent perspective of work stressors by considering the influence of ESM usage on work stressors. The findings indicate that ESM use can have a positive role in overcoming workplace stress such as challenge and hindrance.

Secondly, by integrating individuals' stress assessment methods, we have contributed valuable insights into the transactional theory of stress. According to this theory, stressors can be simultaneously evaluated as both challenges and hindrances; this initial perception serves as the key mechanism connecting stressors to outcomes (Webster et al., 2011). According to the findings, challenge stressors have a positive effect on work efficiency while hindrance stressors have a negative effect on it. These outcomes contribute to the research on stressors and work efficiency by responding to the demand that “various stress events may function via distinct mechanisms to variably impact work efficiency (Byron et al., 2010).”

Thirdly, the findings emphasize the importance of resilience within the context of the organization (Kimura et al., 2018). According to the findings, resilience moderates the association between challenge stressors and work efficiency. The higher the perceived capacity to bounce back, the higher the impact of challenge stressors on work efficiency. This conclusion is compatible with the principle of a transactional theory of stress. Specifically, employees who believe they are more resilient are more confident in overcoming the risk of job demands when confronted with workplace challenges (Zhou et al., 2021). On the other hand, individuals who believe they are low in resilience are less confident in addressing workplace stress.

### Managerial Implications

This research also has numerous managerial implications. Firstly, its findings indicate that using ESM is beneficial for both challenge and hindrance stresses. As a result, managers need to focus on and encourage the use of ESM, which benefit all workers in obtaining task control and addressing the problem of stressful demands. Accordingly, managers may digitize task-related activities and motivate employees to utilize ESM to organize, schedule, and explain project details on the ESM platform. Furthermore, managers can organize ESM-related training, seminars, and conferences to assist employees to learn how to utilize ESM to handle their responsibilities and better manage their jobs. Nevertheless, managers should also implement policies related to ESM usage to control excessive use, since previous studies have also observed the adverse effect of the excessive use of ESM in the workplace (Cao and Yu, 2019; Chen and Wei, 2019).

Secondly, the results showed that challenge stressors have a beneficial impact on work efficiency whereas hindrance stressors have a negative influence. As a result, we propose that managers assist employees to understand the responsibility and workload of their job requirements when faced with problems. However, managers should be careful of hindrance stresses that prevent employees from suggesting task-related solutions. Specifically, managers should reduce workplace hindrance stressors and effectively schedule challenging assignments for their employees on the ESM platform (Kimura et al., 2018). The smallest hindrance stressors may likely create psychological exhaustion and are thus detrimental to employee work efficiency. Adequate challenges can encourage employees to devote more time and attention to their tasks, resulting in increased work efficiency (Wallace et al., 2009).

Thirdly, the findings of this study indicate that a resilient employee may efficiently deal with workplace stressors. This conclusion is practically significant since the resilience evaluated in this research is a skill that can be improved through proper training. Given this, managers must promote employees' resilience as an element of psychological capital that allows employees to efficiently deal with work stressors (Liu et al., 2013; Kimura et al., 2018).

### Limitations and Future Directions

Although the findings of this research have numerous theoretical and managerial implications, they also have certain limitations. These require a “with reservations” approach but also open up several possibilities for further investigation. Firstly, we measured ESM usage as a single construct; however, scholars have recently categorized ESM usage as social- and work-related (Ali-Hassan et al., 2015). Future scholars could thus investigate the impact of ESM usage, both social- and work-related, on individual work efficiency. The application of ESM for social purposes may provide more interesting findings, as social support from coworkers is essential for stressed employees.

Secondly, we emphasized the role of resilience as a moderating element on the connection between work stressors and individual work efficiency. Although resilience is an important work environment factor (Kimura et al., 2018), an examination of the influence of other psychological factors on work efficiency may offer more comprehensive guidance for managerial operations. Future researchers may also use other workplace factors such as employee job security and work motivation (Kim and Beehr, 2018) to investigate the impact of work stressors on employee work efficiency. Furthermore, this study concentrated on the positive impact of ESM usage; however, recent research has emphasized the detrimental impacts of ESM usages such as strain (Pitafi et al., 2020b), overloaded individual outcomes, and on creative performance (Cao and Yu, 2019; Chen and Wei, 2019). As a consequence, future researchers can examine the relationship between the overuse of social media and work efficiency.

Thirdly, since we did cross-sectional research, it is impossible to draw any conclusion about the causal nature of the analyzed associations (Podsakoff et al., 2012). Future longitudinal research may investigate the ability of stressed employees to work efficiency. To further extend the casual relationship of this study, an experimental study may be more suitable. In addition, employee work efficiency was rated by individuals. Previous research has analyzed self-reported work performance in several studies (Cao et al., 2016; Pitafi et al., 2018b). Measuring the work performance from distinct places may also assist in eliminating the possibility of CMV when examining the link between stress and outcome. Therefore, findings based on performance-rated distinct sources may also provide more convincing conclusions.

## Conclusion

The objective of this study was to examine the employee work efficiency in stress environment using survey data collected from China. Findings indicated that current data supports almost all the proposed hypothesis. Specifically, outcome indicated that ESM usage have negative impact on challenge stress and hindrance stress. Challenge stress has shown significant effect on work efficiency whereas hindrance stress have negative impact work efficiency. In addition, results also indicated that resilience significantly moderates the relationship between challenge stress and work efficiency. However, resilience have shown insignificant moderating effect in the relationship between hindrance stress and work efficiency.

## Data Availability Statement

The raw data supporting the conclusions of this article will be made available by the authors, without undue reservation.

## Ethics Statement

The studies involving human participants were reviewed and approved by Hefei University of Technology China. The patients/participants provided their written informed consent to participate in this study.

## Author Contributions

All authors listed have made a substantial, direct and intellectual contribution to the work, and approved it for publication.

## Conflict of Interest

The authors declare that the research was conducted in the absence of any commercial or financial relationships that could be construed as a potential conflict of interest.

## Publisher's Note

All claims expressed in this article are solely those of the authors and do not necessarily represent those of their affiliated organizations, or those of the publisher, the editors and the reviewers. Any product that may be evaluated in this article, or claim that may be made by its manufacturer, is not guaranteed or endorsed by the publisher.
